# Global Farm Animal Production and Global Warming: Impacting and Mitigating Climate Change

**DOI:** 10.1289/ehp.11034

**Published:** 2008-01-31

**Authors:** Gowri Koneswaran, Danielle Nierenberg

**Affiliations:** 1 Humane Society of the United States, Washington, DC, USA; 2 Worldwatch Institute, Washington, DC, USA

**Keywords:** animal agriculture, CAFO, climate change, concentrated animal feeding operation, diet, environment, farm animals, farm animal welfare, food choices, global warming, greenhouse gas emissions (GHGs)

## Abstract

**Background:**

The farm animal sector is the single largest anthropogenic user of land, contributing to many environmental problems, including global warming and climate change.

**Objectives:**

The aim of this study was to synthesize and expand upon existing data on the contribution of farm animal production to climate change.

**Methods:**

We analyzed the scientific literature on farm animal production and documented greenhouse gas (GHG) emissions, as well as various mitigation strategies.

**Discussions:**

An analysis of meat, egg, and milk production encompasses not only the direct rearing and slaughtering of animals, but also grain and fertilizer production for animal feed, waste storage and disposal, water use, and energy expenditures on farms and in transporting feed and finished animal products, among other key impacts of the production process as a whole.

**Conclusions:**

Immediate and far-reaching changes in current animal agriculture practices and consumption patterns are both critical and timely if GHGs from the farm animal sector are to be mitigated.

Although much evidence has been amassed on the negative impacts of animal agricultural production on environmental integrity, community sustainability, public health, and animal welfare, the global impacts of this sector have remained largely underestimated and underappreciated. In a recent review of the relevant data, Steinfeld et al. (2006) calculated the sector’s contributions to global greenhouse gas (GHG) emissions and determined them to be so significant that—measured in carbon dioxide equivalent—the emissions from the animal agricultural sector surpass those of the transportation sector.

## Global warming and climate change

The three main GHGs are CO_2_, methane (CH_4_), and nitrous oxide (N_2_O) (Steinfeld et al. 2006). Although most attention has focused on CO_2_, methane and N_2_O—both extremely potent GHGs—have greater global warming potentials (GWPs) than does CO_2_. By assigning CO_2_ a value of 1 GWP, the warming potentials of these other gases can be expressed on a CO_2_-equivalent basis (Paustian et al. 2006; Steinfeld et al. 2006): CH_4_ has a GWP of 23, and N_2_O has a GWP of 296.

Many impacts of global warming are already detectable. As glaciers retreat, the sea level rises, the tundra thaws, hurricanes and other extreme weather events occur more frequently, and penguins, polar bears, and other species struggle to survive (Topping 2007), experts anticipate even greater increases in the intensity and prevalence of these changes as the 21st century brings rises in GHG emissions. The five warmest years since the 1890s were 1998, 2002, 2003, 2004, and 2005 [NASA (National Aeronautics and Space Administration) 2006]. Indeed, average global temperatures have risen considerably, and the Intergovernmental Panel on Climate Change (IPCC 2007c) predicts increases of 1.8–3.9°C (3.2–7.1°F) by 2100. These temperature rises are much greater than those seen during the last century, when average temperatures rose only 0.06°C (0.12°F) per decade (National Oceanic and Atmospheric Administration 2007). Since the mid-1970s, however, the rate of increase in temperature rises has tripled. The IPCC’s latest report (IPCC 2007b) warns that climate change “could lead to some impacts that are abrupt or irreversible.”

## Anthropogenic influences

Although some natural occurrences contribute to GHG emissions (IPCC 2007c), the overwhelming consensus among the world’s most reputable climate scientists is that human activities are responsible for most of this increase in temperature (IPCC 2007a). The IPCC (2007a) concluded

with high confidence that anthropogenic warming over the last three decades has had a discernible influence on many physical and biological systems.

Although transportation and the burning of fossil fuels have typically been regarded as the chief contributors to GHG emissions and climate change, a 2006 report, *Livestock’s Long Shadow: Environmental Issues and Options* [Food and Agriculture Organization of the United Nations (FAO) 2006], highlighted the substantial role of the farm animal production sector. Identifying it as “a major threat to the environment” (FAO 2006), the FAO found that the animal agriculture sector emits 18%, or nearly one-fifth, of human-induced GHG emissions, more than the transportation sector. (Steinfeld et al. 2006).

Our objective was to outline the animal agriculture sector’s share of global GHG emissions by synthesizing and expanding upon the data reported in *Livestock’s Long Shadow* (FAO 2006) with more recent reports from the IPCC, data from the U.S. Environmental Protection Agency (EPA), and studies on GHGs from agriculture and mitigation strategies [Cederberg and Stadig 2003; International Federation of Organic Agriculture Movements (IFOAM) 2004; IPCC 2007a, 2007b, 2007c; McMichael et al. 2007; Ogino et al. 2007; U.S. EPA 2007a; Verge et al. 2007]. We also investigated links between this sector and the far-reaching impacts of climate change on conflict, hunger, and disease, while underscoring the roles of animal agriculture industries, policy makers, and individual consumers in mitigating this sector’s contributions to climate change and global warming.

## Discussion

### Impacts of growing livestock populations and intensifying production

According to FAOSTAT (FAO 2008), globally, approximately 56 billion land animals are reared and slaughtered for human consumption annually, and livestock inventories are expected to double by 2050, with most increases occurring in the developing world (Steinfeld et al. 2006). As the numbers of farm animals reared for meat, egg, and dairy production rise, so do their GHG emissions. The U.S. Department of Agriculture (USDA 2004) has noted that

GHG emissions from livestock are inherently tied to livestock population sizes because the livestock are either directly or indirectly the source for the emissions.

Since the 1940s, for example, escalating farm animal populations—in large, confined operations, in particular—have significantly increased methane emissions from both animals and their manure (Paustian et al. 2006).

In recent decades, increasing numbers of animals are raised in intensive production systems in which chickens, pigs, turkeys, and other animals are confined in cages, crates, pens, stalls, and warehouse-like grow-out facilities. These production systems are devoid of environmental stimuli, adequate space, or means by which to experience most natural behaviors. Furthermore, because these industrialized, “landless” facilities tend to produce more manure than can be used as fertilizer on nearby cropland (FAO 2005b), manure is instead “distributed to a small, local landmass resulting in soil accumulation and runoff of phosphorus, nitrogen, and other pollutants” (Thorne 2007).

Although extensive or pasture-based farming methods remain the norm in Africa and some parts of Asia, the trend in Latin America and Asia is to increasingly favor intensive production systems over more sustainable and more animal welfare–friendly practices (Nierenberg 2006). According to a 2007 report describing GHG emissions from agriculture (Verge et al. 2007),

In recent years, industrial livestock production has grown at twice the rate of more traditional mixed farming systems and at more than six times the rate of production based on grazing.

Confining greater numbers of animals indoors and further separating production operations from agricultural land will only exacerbate the environmental problems already posed by this sector, which the FAO has deemed “one of the top two or three most significant contributors to the most serious environmental problems, at every scale from local to global” (Steinfeld et al. 2006).

### CO_2_ emissions from animal agriculture

Regarded as the most important GHG, CO_2_ has the most significant direct-warming impact on global temperature because of the sheer volume of its emissions. Of all the natural and human-induced influences on climate over the past 250 years, the largest is due to increased CO_2_ concentrations attributed to burning fossil fuels and deforestation (Bierbaum et al. 2007).

The animal agriculture sector accounts for approximately 9% of total CO_2_ emissions, which are primarily the result of fertilizer production for feed crops, on-farm energy expenditures, feed transport, animal product processing and transport, and land use changes (Steinfeld et al. 2006).

Burning fossil fuels to produce fertilizers for feed crops may emit 41 million metric tons of CO_2_ per year (Steinfeld et al. 2006). Vast amounts of artificial nitrogenous fertilizer are used to grow farm animal feed, primarily composed of corn and soybeans. Most of this fertilizer is produced in factories dependent on fossil-fuel energy (Steinfeld et al. 2006). The Haber-Bosch process, which produces ammonia in order to create nitrogen-based artificial fertilizer, is used to produce 100 million metric tons of fertilizer for feed crops annually (Steinfeld et al. 2006).

An additional 90 million metric tons of CO_2_ per year may be emitted by fossil fuels expended for intensive confinement operations (Steinfeld et al. 2006). Energy uses in these industrial facilities differ substantially from those in smaller-scale, extensive, or pasture-based farms. Although a large portion of the energy used for intensive confinement operations goes toward heating, cooling, and ventilation systems, more than half is expended by feed crop production, specifically to produce seed, herbicides, and pesticides, as well as the fossil fuels used to operate farm machinery in the production of feed crops (Steinfeld et al. 2006).

According to the FAO’s estimates, CO_2_ emissions from farm animal processing total several tens of millions of metric tons per year (Steinfeld et al. 2006). The amount of fossil fuels burned varies depending on the species and type of animal product. For example, processing 1 kg of beef requires 4.37 mega-joules (MJ), or 1.21 kilowatt-hours, and processing 1 dozen eggs requires > 6 MJ, or 1.66 kilowatt-hours (Steinfeld et al. 2006).

That same 1 kg of beef may result in GHGs equivalent to 36.4 kg of CO_2_, with almost all the energy consumed attributed to the production and transport of feed (Ogino et al. 2007). Approximately 0.8 million metric tons of CO_2_ are emitted annually from the transportation of feed and animal products to the places where they will be consumed (Steinfeld et al. 2006).

Farm animals and animal production facilities cover one-third of the planet’s land surface, using more than two-thirds of all available agricultural land including the land used to grow feed crops (Haan et al. 1997). Deforestation, land degradation, soil cultivation, and desertification are responsible for CO_2_ emissions from the livestock sector’s use of land.

Animal agriculture is a significant catalyst for the conversion of wooded areas to grazing land or cropland for feed production, which may emit 2.4 billion metric tons of CO_2_ annually as a result of deforestation (Steinfeld et al. 2006). This sector has particularly devastated Latin America, the region experiencing the largest net loss of forests and greatest releases of stored carbon into the atmosphere, resulting from disappearing vegetation (Steinfeld et al. 2006). One of the chief causes of Latin America’s deforestation is cattle ranching (FAO 2005a).

Other important ecosystems are also threatened by increasing farm animal populations. Brazil’s Cerrado region, the world’s most biologically diverse savannah, produces half of the country’s soy crops [Klink and Machado 2005; World Wildlife Fund (WWF) 2007a, 2007b]. As noted by the WWF (2007a), the region’s animal species

are competing with the rapid expansion of Brazil’s agricultural frontier, which focuses primarily on soy and corn. Ranching is another major threat to the region, as it produces almost 40 million cattle a year.

Farm animal production also results in releases of up to 28 million metric tons of CO_2_/year from cultivated soils (Steinfeld et al. 2006). Soils, like forests, act as carbon sinks and store more than twice the carbon found in vegetation or in the atmosphere (Steinfeld et al. 2006). Human activities, however, have significantly depleted the amount of carbon sequestered in the soil, contributing to GHG emissions (Steinfeld et al. 2006).

Desertification, or the degradation of land in arid, semiarid, and dry subhumid areas, is also exacerbated and facilitated by the animal agriculture sector (FAO 2007). By reducing the productivity and amount of vegetative cover, desertification allows CO_2_ to escape into the atmosphere. Desertification of pastures due to animal agriculture is responsible for up to 100 million metric tons of CO_2_ emissions annually (Steinfeld et al. 2006).

### Nitrogen from fertilizer and feed production

Feeding the global population of livestock requires at least 80% of the world’s soybean crop and more than one-half of all corn (Ash M, Nierenberg D, personal communication; Halweil B, Smil V, personal communication), a plant whose growth is especially dependent on nitrogen-based artificial fertilizers. Natural sources of fixed nitrogen, the form easily available as fertilizer for plants, are limited, necessitating artificial fertilizer production. Before the development of the Haber-Bosch process, the amount of sustainable life on Earth was restricted by the amount of nitrogen made available to plants by bacteria and lightning. Modern fertilizer manufacturing, heavily reliant on fossil fuels, has taken a once-limited nutrient and made it available in massive quantities for crop farmers in the industrialized world and, increasingly, the developing world.

According to Elizabeth Holland, a senior scientist with the National Center for Atmospheric Research (Bohan 2007),

The changes to the nitrogen cycle are larger in magnitude and more profound than the changes to the carbon cycle. . . . But the nitrogen cycle is being neglected.

In addition, the co-chairs of the Third International Nitrogen Conference highlighted the role of farm animal production in the Nanjing Declaration on Nitrogen Management (Zhu et al. 2004), a statement presented to the United Nations Environment Programme, recognizing that

a growing proportion of the world’s population consumes excess protein and calories, which may lead to human health problems. The associated production of these dietary proteins (especially animal products) leads to further disturbance of the nitrogen cycle.

According to Vaclav Smil, a nitrogen cycle expert at the University of Manitoba, “we have perturbed the global nitrogen cycle more than any other, even carbon” (Pollan 2006). Indeed, the overwhelming majority of all crops grown in the industrialized world are nitrogen-saturated, and overuse of nitrogen in crop production, nitrogen runoff into waterways, and the millions of tons of nitrogen found in farm animal manure threaten environmental integrity and public health.

### Methane and N_2_O

The animal agriculture sector is also responsible for 35–40% of annual anthropogenic methane emissions (Steinfeld et al. 2006) that result from enteric fermentation in ruminants and from farm animal manure. Methane emissions are affected by a number of factors, including the animal’s age, body weight, feed quality, digestive efficiency, and exercise (Paustian et al. 2006; Steinfeld et al. 2006).

Ruminants emit methane as part of their digestive process, which involves microbial (enteric) fermentation (Steinfeld et al. 2006; U.S. EPA 2006). Although individual animals produce relatively small amounts of methane (U.S. EPA 2007b), the > 1 billion ruminants reared annually amount to a significant methane source (FAO 2008). Indeed, enteric fermentation generates approximately 86 million metric tons of methane emissions worldwide (Steinfeld et al. 2006).

Typically, cattle confined in feedlots or in intensive confinement dairy operations are fed an unnatural diet of concentrated high-protein feed consisting of corn and soybeans. Although cattle may gain weight rapidly when fed this diet (Pollan 2002), it can cause a range of illnesses (Smith 1998). This diet may also lead to increased methane emissions. The standard diet fed to beef cattle confined in feedlots contributes to manure with a “high methane producing capacity” (U.S. EPA 1998). In contrast, cattle raised on pasture, eating a more natural, low-energy diet composed of grasses and other forages, produce manure with about half of the potential to generate methane (U.S. EPA 1998).

Farm animals produce billions of tons of manure, with confined farm animals in the United States alone generating approximately 500 million tons of solid and liquid waste annually (U.S. EPA 2003). Storing and disposing of these immense quantities of manure can lead to significant anthropogenic emissions of methane and N_2_O (U.S. EPA 2007a). For example, according to the Pew Center on Global Climate Change (Paustian et al. 2006), farm animal manure management accounts for 25% of agricultural methane emissions in the United States and 6% of agricultural N_2_O emissions. Globally, emissions from pig manure alone account for almost half of all GHG emissions from farm animal manure (Steinfeld et al. 2006).

Farm animal manure is the source of almost 18 million metric tons of annual methane emissions (Steinfeld et al. 2006). Between 1990 and 2005 in the United States, methane emissions from dairy cow and pig manure rose by 50% and 37%, respectively (U.S. EPA 2007a). The U.S. EPA (2007a) traces this increase to the trend toward housing dairy cows and pigs in larger facilities that typically use liquid manure management systems, which were first in use in the 1960s (Miner et al. 2000) but are now found in large dairy operations across the United States and in some developing countries, as well as in most industrial pig operations worldwide.

Although 70% of anthropogenic emissions of N_2_O result from crop and animal agriculture combined, farm animal production, including growing feed crops, accounts for 65% of global N_2_O emissions (Steinfeld et al. 2006). Manure and urine from farm animals, once deposited on the soil, emit N_2_O; in the United States, a 10% rise in N_2_O emissions between 1990 and 2005 can be traced, in part, to changes in the poultry industry, including an overall increase in the domestic stock of birds used for meat and egg production (U.S. EPA 2007a).

### Conflict, hunger, and disease

As is the case with animal agriculture’s impacts on soil, water, and air quality, the sector’s contributions to climate change cannot be viewed in a vacuum. Climate change is having far-reaching consequences, perhaps most startlingly seen in growing conflicts among pastoral communities. Environmental degradation has been cited as one of the catalysts for ongoing conflicts in Darfur and other areas of Sudan [United Nations Environment Programme (UNEP) 2007], where the effects of climate change have led to untenable conditions. As temperatures rise and water supplies dry up, farmers and herders are fighting to gain and control diminishing arable land and water (Baldauf 2006).

The UNEP (2007) tied two of its critical concerns in Sudan—land degradation and desertification—to “an explosive growth in livestock numbers.” In addition to citing climate change as one factor that led to the Darfur conflict (Ban 2007), United Nations Secretary-General Ban Ki-moon has noted that natural disasters, droughts, and other changes brought about by global warming “are likely to become a major driver of war and conflict” (United Nations 2007).

According to the IPCC (2007a), many areas already suffering from drought will become drier, exacerbating the risks of both hunger and disease. By 2020, up to 250 million people may experience water shortages, and, in some countries, food production may be cut in half (IPCC 2007a). By 2050—the same year by which the FAO projects that meat and dairy production will double from present levels, primarily in the developing world (Steinfeld et al. 2006)—130 million people in Asia may suffer from climate-change–related food shortages (Casey 2007).

Global temperature shifts may also hasten the speed at which infectious diseases emerge and reemerge (Epstein and Mills 2005). According to Francois Meslin of the World Health Organization, “the chief risk factor for emerging zoonotic diseases is environmental degradation by humans, particularly deforestation, logging, and urbanisation” (Fleck 2004). The clear-cutting of forests for soybean cultivation, logging, and other industries enables viruses to exploit such newly exposed niches (Greger 2007).

### Strategies and next steps

Mitigating the animal agriculture sector’s contributions to climate change necessitates comprehensive and immediate action by policy makers, producers, and consumers. Enhanced regulation is required in order to hold facilities accountable for their GHG emissions. One critical step is accurately pricing environmental services—natural resources that are typically free or underpriced—leading to “overexploitation and pollution” (Steinfeld et al. 2006).

Thus far, most mitigation and prevention strategies undertaken by the animal agriculture sector have focused on technical solutions. For example, researchers are investigating the reformulation of ruminant diets to reduce enteric fermentation and some methane emissions (Connolly 2007). One such remedy is a plant-based bolus, formulated to reduce excessive fermentation and regulate the metabolic activity of rumen bacteria to reduce methane emissions from both the animals and their manure (Drochner W, Nierenberg D, personal communication).

The USDA and U.S. EPA assist in funding anaerobic digester projects domestically and abroad (U.S. EPA 2007c; Sutherly 2007). These digesters, now in use at some large-scale intensive confinement facilities, capture methane from manure to use as a source of energy (Storck 2007), but are typically not economically viable for small-scale farms (Silverstein 2007).

In addition, producers are burning animal waste for fuel. The world’s foremost pig producer, Smithfield Foods (Smithfield, VA), and one of the top poultry producers, Tyson Foods (Springdale, AR), are both using animal by-product fats to create biofuels (Johnston 2007; PR Newswire 2007).

McDonald’s (Oak Brook, IL) and agri-business giant Cargill (Wayzata, MN), which was supplying McDonald’s with soy for use as chicken feed, recently entered into an agreement with Brazil’s other chief soy traders. Engineered by international environmental organization Greenpeace, a 2-year moratorium was enacted in 2007 to prevent purchases of soy from Brazil’s newly deforested areas (Kaufman 2007).

As consumers increasingly favor more environmentally friendly products and techniques, reducing consumption of meat, eggs, and milk, as well as choosing more sustainably produced animal products, such as those from organic systems, may prove equally critical strategies. Indeed, organic farming has the potential to reduce GHG emissions and sequester carbon (IFOAM 2004). Also, raising cattle for beef organically on grass, in contrast to fattening confined cattle on concentrated feed, may emit 40% less GHGs and consume 85% less energy than conventionally produced beef (Cederberg and Stadig 2003; Fanelli 2007; Ogino et al. 2007).

However, there remains an immediate need for more research regarding both technical and less technology-dependent strategies to record existing GHG emissions from individual production facilities and to provide lessons to producers and policy makers for reducing the climate-damaging impacts of animal agriculture.

Given the urgency for global action—calls echoed by scientists and world leaders alike—individual consumers must also participate. McMichael et al. (2007) put forth several recommendations, including the reduction of meat and milk intake by high-income countries as “the urgent task of curtailing global greenhouse-gas emissions necessitates action on all major fronts”; they concluded that, for high-income countries, “greenhouse-gas emissions from meat-eating warrant the same scrutiny as do those from driving and flying.”

## Conclusion

As the numbers of farm animals reared for meat, egg, and dairy production increase, so do emissions from their production. By 2050, global farm animal production is expected to double from present levels. The environmental impacts of animal agriculture require that governments, international organizations, producers, and consumers focus more attention on the role played by meat, egg, and dairy production. Mitigating and preventing the environmental harms caused by this sector require immediate and substantial changes in regulation, production practices, and consumption patterns.
